# Above-Knee Amputation in the Setting of a Chronic Soft Tissue Infection and Periprosthetic Joint Infection With Exposed Hardware

**DOI:** 10.7759/cureus.99063

**Published:** 2025-12-12

**Authors:** Kiana Bennett, Srikanth A Pillai, William F Oppat

**Affiliations:** 1 Vascular Surgery, Henry Ford Providence Hospital, Southfield, USA; 2 Vascular Surgery, Wayne State University School of Medicine, Detroit, USA

**Keywords:** above knee amputation, chronic osteomyelitis, exposed knee hardware, periprosthetic joint infection, total knee arthroplasty

## Abstract

Total knee arthroplasty is one of the most common and successful orthopedic surgeries for the treatment of osteoarthritis. While infection is a common postoperative complication, periprosthetic joint infection is a rarer type of infection requiring aggressive treatment. This case report reviews the clinical course of a 71-year-old female with a history of recurrent prosthetic joint infections and osteomyelitis of the left knee and femur following a left total knee arthroplasty. Ten years after the index procedure, she developed a soft tissue ulcer and non-healing wound that led to exposure of her knee joint hardware at the level of the skin. Due to her complicated medical history, it was decided she would undergo an above-knee amputation. This report discusses a unique patient history of recurrent periprosthetic joint infections requiring extensive surgical and medical management, ultimately leading to an above-knee amputation. It also highlights the importance of a multidisciplinary team in the setting of complex patient care.

## Introduction

Overall life expectancy continues to improve as total knee arthroplasty (TKA) has become a mainstay of treatment for debilitating osteoarthritis. Prosthetic joint infection (PJI) is a major cause of prosthetic failure after TKA with an estimated incidence between 0.5-2% [1]. It is associated with substantial morbidity, mortality, and cost [2]. Management of PJI involves a multidisciplinary approach including surgical revision, debridement, and antimicrobial therapy [3]. However, when multiple revisions and antibiotics fail to eradicate the infection, above-knee amputation (AKA) may be used as a salvage option [4]. Amputation allows patients to regain some mobility with the option for a prosthetic. Overall studies show patient satisfaction following AKA in this context is as high as 85% [5,6]. This remains a high-risk procedure in a vulnerable patient population with a 5-year mortality estimates as high as 50% [6].

This case discusses the complex history of a 71-year-old female who underwent a TKA that led to the development of multiple PJIs requiring a series of surgical interventions. Her care was closely monitored by the infectious diseases department, wound care clinic, and the orthopedic department with eventual involvement of the vascular surgery department. Ten years after her initial surgery, she developed a large posterior thigh pressure ulcer and a non-healing left knee wound with exposed hardware. After failed surgical and medical management and decreased quality of life, it was decided that she should undergo an AKA. This case aims to highlight the multidisciplinary approach to the management of chronic and complex PJIs, as well as present a unique case.

## Case presentation

A 71-year-old female with a medical history significant for morbid obesity, recurrent renal calculi, neurogenic bladder, periprosthetic left femur fracture, recurrent osteomyelitis/PJI of the left knee and femur, and bilateral lower extremity chronic lymphedema presented to the emergency department (ED) following an outpatient visit with the infectious disease department. She has a complex history with the orthopedic and infectious disease departments following her TKA in 2013. Notable surgical history is a left TKA and ureteral stent placements, cystoscopy, and lithotripsy for recurrent renal calculi. Her social history notes that she lives in a skilled nursing facility requiring assistance with most activities of daily living. She has been non-ambulatory for over eight years.

She had a left TKA in 2013, and two weeks postoperatively, she had a periprosthetic femur fracture that required a secondary reconstruction with a distal femoral replacement. In mid 2014, she had presented to the ED for persistent knee pain following the secondary reconstruction. Arthrocentesis was done at the time, which grew two strains of coagulase-negative *Staphylococcus epidermidis*. During that time, she underwent a resection arthroplasty with placement of an antibiotic spacer and immobilization rod, followed by six weeks of IV vancomycin and then oral minocycline. Upon completion of her antibiotics, she underwent a revision arthroplasty with placement of a hinge prosthetic with postoperative IV vancomycin for four weeks due to her history of a previous infection.

**Figure 1:**
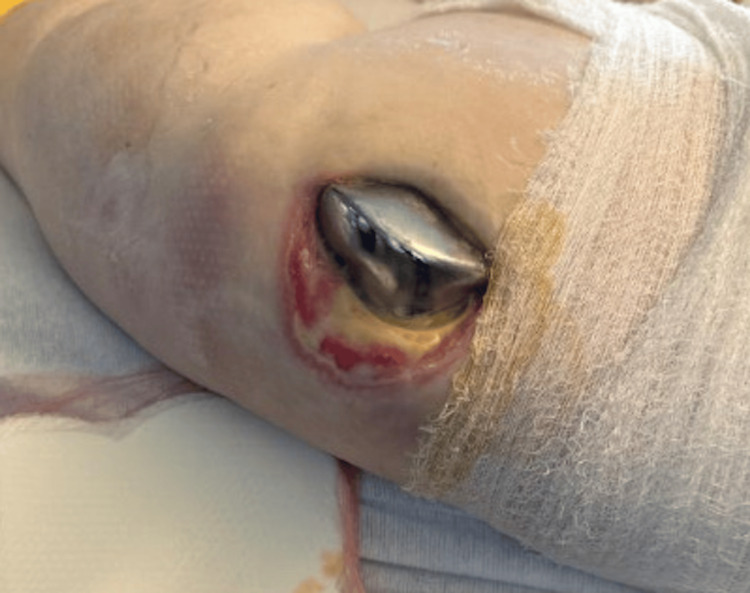
Left knee exposed joint hardware Exposed left knee joint hardware following a non-healing wound.

**Figure 2:**
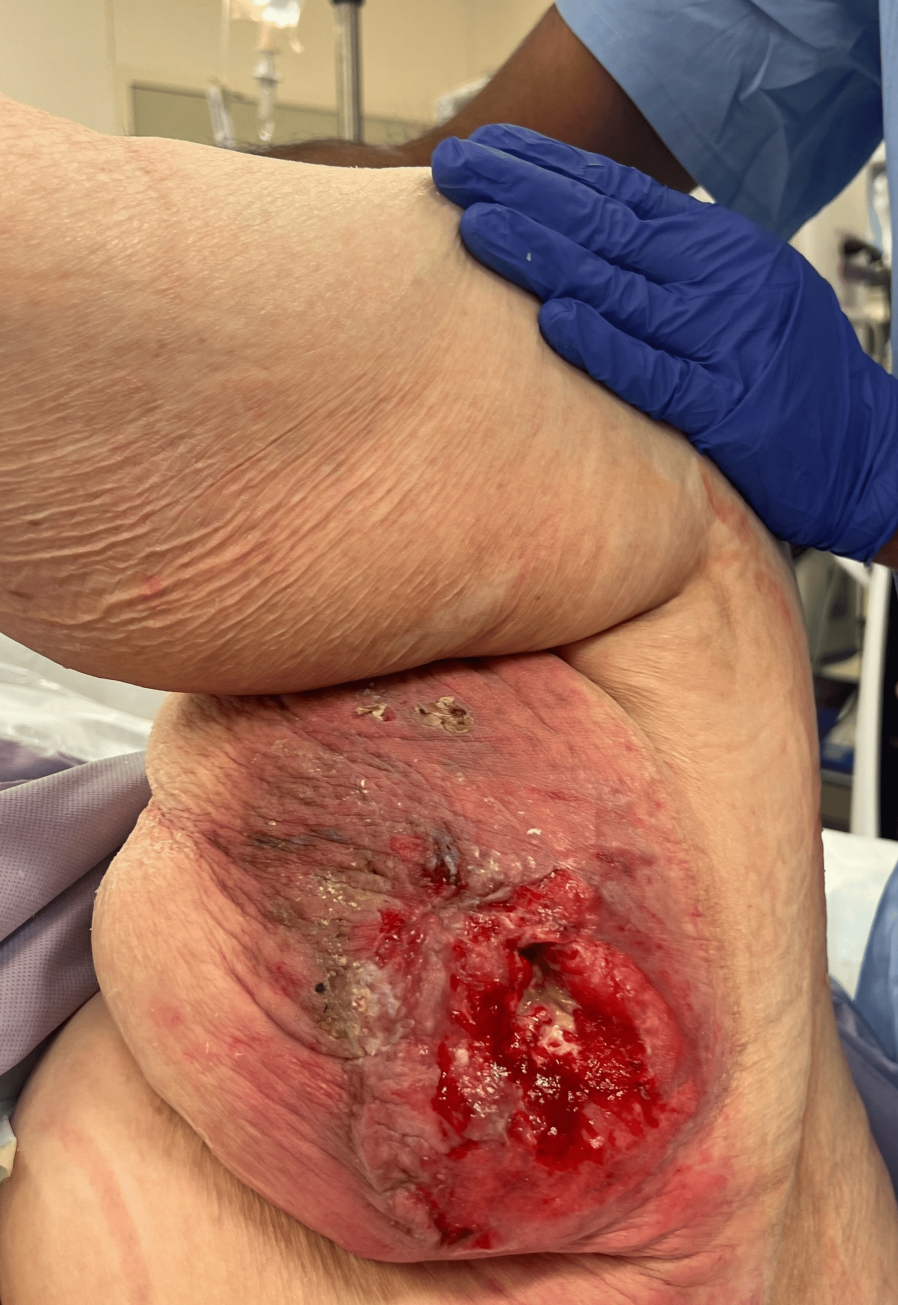
Left posterior thigh pressure ulcer Superficial pressure ulcer on the posterior thigh. This was taken after approximately 10 months of wound care management and antibiotics.

**Figure 3:**
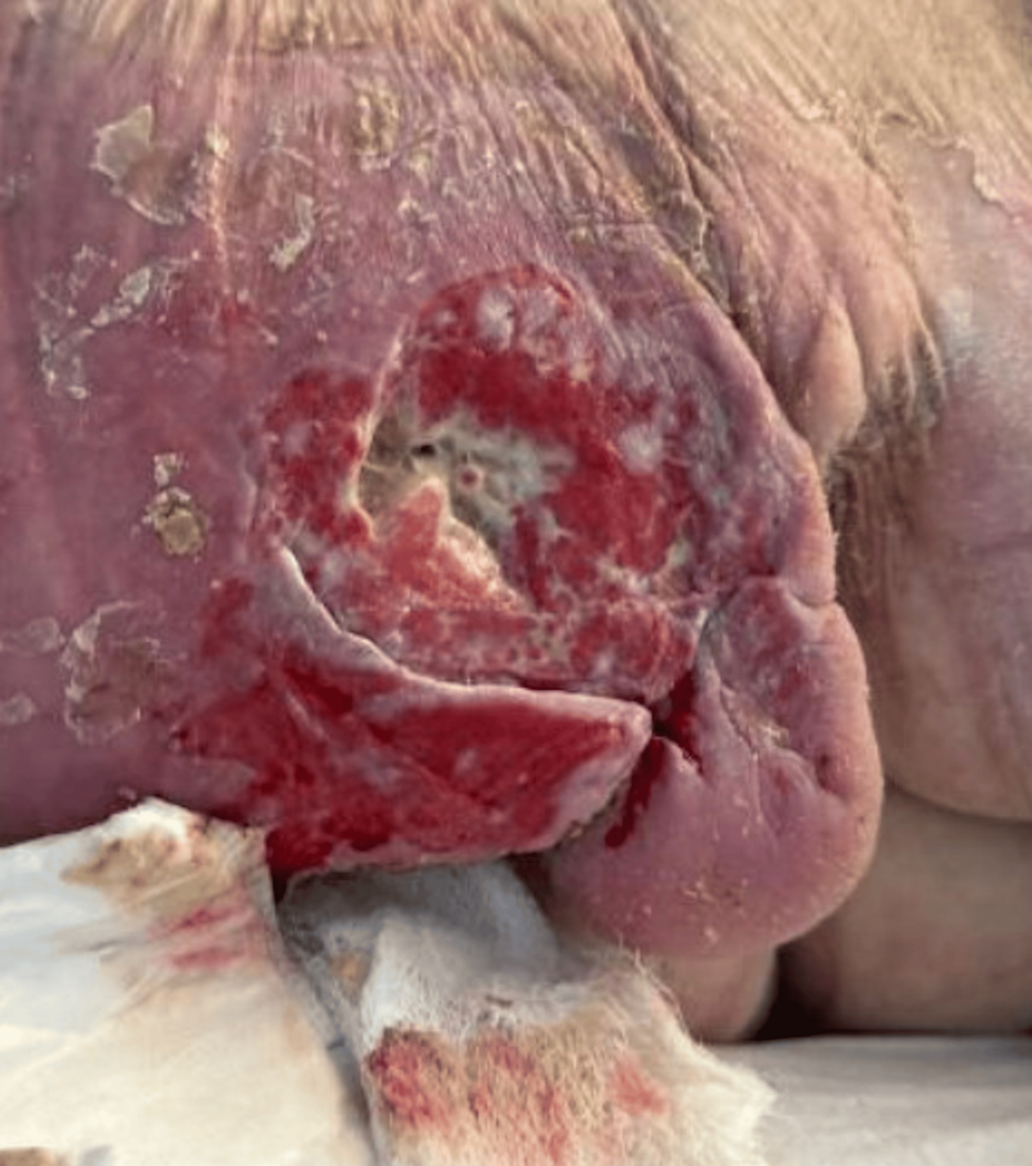
Left posterior thigh ulcer Magnified view of the pressure ulcer seen on the left posterior thigh.

**Figure 4:**
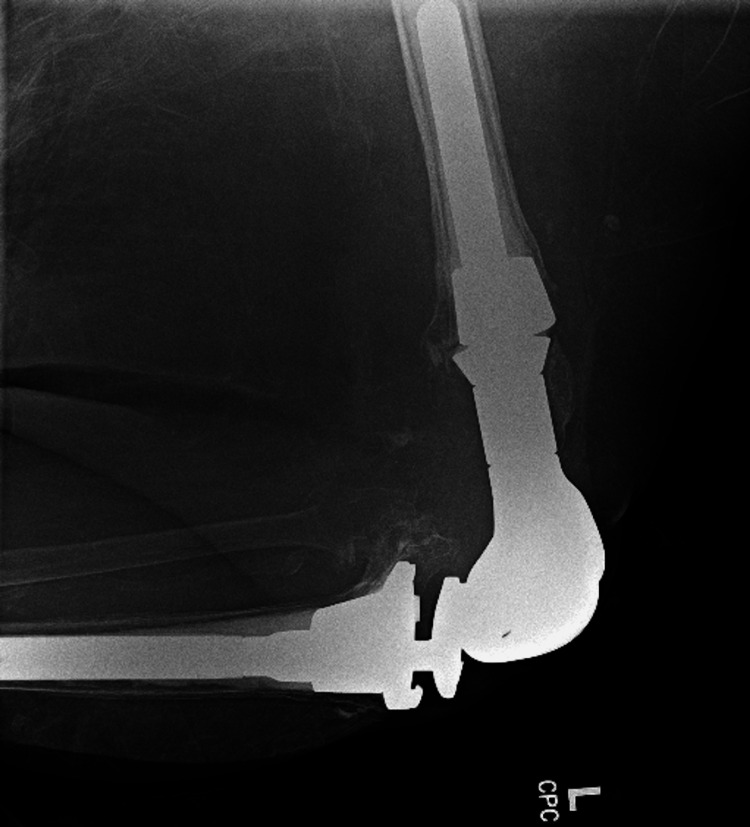
Left knee X-ray This X-ray highlights the components of the knee joint. The hinged knee joint prosthesis that inserts into the femur. A long stem prosthesis is seen in the tibia.

**Figure 5:**
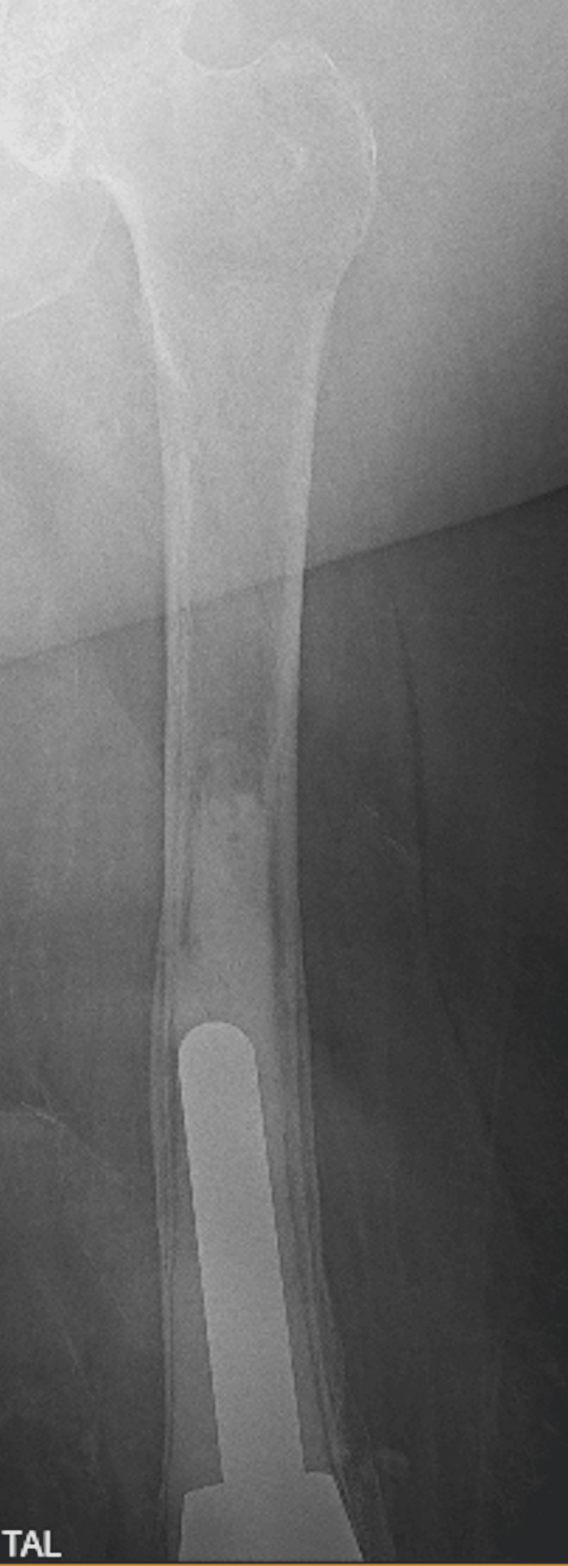
Left femur X-ray This image shows how proximal the femoral hinge joint extends into the bone.

In early 2024, she presented to the ED from the wound care clinic due to a non-healing left knee wound with exposed knee joint hardware (Figure 1). Cultures at that time grew *Staphylococcus lugdunensis,* and she was treated appropriately. She was then started on daily prophylactic antibiotics with doxycycline and 12 weeks of IV dalbavancin, which was managed by the infectious disease department. Wound care had also been following her for a pressure ulcer on the left posterior medial thigh (Figures 2, 3). By late 2024, her posterior medial thigh wound showed minimal improvement with oral and IV antibiotics. She was then referred to the ED for IV antibiotics and consideration of more aggressive treatment versus an above-knee amputation (AKA). Another culture was taken of the exposed hardware due to the purulent drainage seen at the left knee, which grew *Pseudomonas aeruginosa*. She was seen by the orthopedics department who offered continued conservative management, resection arthroplasty, or a high AKA. The vascular surgery department was consulted as the patient would likely undergo an AKA. She had a knee and femur X-ray to assist with preoperative planning, to further assess how high the amputation should be, with consideration to the existing hardware (Figures 4, 5). After evaluation of the imaging, it was felt that the amputation did not need to be higher than the femoral prosthetic component. During this time, a computer tomography (CT) without contrast of the left lower extremity was done to rule out an abscess of the soft tissue wound. The imaging was unremarkable for any sign of abscess.

The decision-making process for this patient was complex and multifactorial. Both the patient and her family expressed concerns that the complications following her knee arthroplasty in 2013 have greatly limited her mobility and independence. Possible amputation had been previously discussed at length with the patient at prior office visits with multiple specialties. The vascular department had a discussion of a below-knee transection, as this would give the best results in terms of mobility, but this was complicated by the posterior left thigh soft tissue infection. A hip disarticulation would eliminate both sources of infection; however, it would greatly limit mobility as she would be unable to have a prosthetic. The orthopedics department had discussed a resection arthroplasty as she already had three revision surgeries, and the joint was unsalvageable. However, a resection arthroplasty would leave the leg with less support, affecting her quality of life and independence. She had undergone months of IV antibiotics to treat the pressure ulcer with the hope of providing healthy tissue to create an uncontaminated flap for an AKA. Despite antibiotic treatment and follow-up with infectious diseases, there was minimal clinical improvement. Due to her limited mobility and presence of infection, it was decided she would undergo an AKA, despite the high risk of wound infection during recovery.

The left AKA was completed alongside the orthopedic surgery team, who removed the prosthetic components. Intraoperative wound cultures from the left posterior infection revealed methicillin-resistant *Staphylococcus aureus* (MRSA). Due to the high risk for wound infection during closure, only the fascial layer was closed. It was decided that the skin would remain open and heal by secondary intention with a wound vacuum placed over the stump. Postoperatively, the stump was healing as expected, and appropriate antibiotics were continued. It was anticipated that her recovery would be complicated by the presence of remaining infection, and the healing process would be variable. On postoperative day 19, she underwent debridement and delayed primary closure of the stump with placement of incisional wound vacuum. She was then discharged to a subacute rehab facility on postoperative day 2 from her delayed closure. However, she was readmitted two days later for superficial wound dehiscence and underwent another debridement. Her wound culture grew MRSA, which was consistent with the intraoperative culture results. Her antibiotics were monitored by the infectious disease department. There was a discussion regarding another debridement in the operating room, but ultimately it was managed conservatively with the placement of a wound vacuum. She was then discharged on postoperative day 10 from her last operative debridement. Since her last discharge, she has not been readmitted for any acute infection.

## Discussion

The most identified microbe in the setting of PJIs is coagulase-negative staphylococcus, specifically *Staphylococcus epidermidis*, followed by *Staphylococcus aureus*, *enterococcus*, and then gram-negative pathogens [7]. *Pseudomonas* is a less common microorganism of PJIs and is a challenging organism to treat and prevent [8]. This pathogen typically attaches to bone and fibrocartilaginous articular surfaces making osteomyelitis and septic arthritis a more common presentation compared to PJI [8]. The presence of *Pseudomonas* is more likely in patients with a history of diabetes mellitus, gastrointestinal or genitourinary surgery [8]. In this case, she has a complex history with multiple renal calculi requiring stents, cystoscopy, and lithotripsy, likely contributing to the presence of this microbe.

PJIs are a rare complication of arthroplasty with an incidence of 1-2% [9]. Treatment options are highly individualized and require a multi-disciplinary team to give the best outcome [10]. Common management of PJIs is antibiotic therapy alongside surgical options such as debridement and implant retention [10]. The surgical gold standard of treatment has been a two-stage exchange [11]. This involves removing the implant followed by IV antibiotics, placement of a temporary spacer, and then reimplantation of the components after the infection has cleared [11]. In some cases, depending on the patient’s comorbidities and presentations, a one-stage or one-and-a-half-stage exchange is becoming a more available treatment option [11]. In this case, the patient had undergone a two-stage revision following her initial PJI with aggressive antibiotics following the surgery. Table 1 illustrates the patient's entire clinical course in chronological order with associated treatment.

**Table 1:** Surgical history and infection timeline This table illustrates the chronological order of the patient's surgical history, treatment, and microbiology history. TKA: Total knee arthroplasty; PJI: Prosthetic joint infection; AKA: Above-knee amputation; MRSA: Methicillin-resistant Staphylococcus aureus.

Year	Clinical course description	Microbiology	Treatment
August 2013	Left TKA		
August 2013 (2 weeks after index procedure)	Periprosthetic left femur fracture		Secondary reconstruction with distal femoral replacement
July-August 2014	PJI	Two strains of *Staphylococcus epidermidis*	Two-stage revision with six weeks of IV vancomycin and four to six weeks of oral minocycline
December 2014	Revision arthroplasty		Four weeks of IV vancomycin in the setting of previous PJI
January 2024	Non-healing left knee wound with exposed hardware and left posterior thigh soft tissue wound	*Staphylococcus lugdunensis* (left knee wound)	Wound care dressing changes. 12 weeks of IV dalbavancin. Close follow-up with the infectious disease department.
October 2024	Non-healing left knee wound with exposed hardware and minimally improved left posterior thigh soft tissue wound.	*Pseudomonas aeruginosa* (left knee wound) and *MRSA* (left posterior thigh soft tissue wound)	High AKA with postoperative IV vancomycin

The patient’s care had been closely followed by multiple specialties to ensure proper management of her infections. However, she had undergone a series of revisions with minimal improvement to her quality of life. Multiple factors were considered before, during, and after surgery to give her the best chance of regaining some level of function and independence. These included (1) the patient’s personal goals regarding her ability to walk and regain some independence, (2) the necessity for a high transection at the thigh given the presence of hardware in the femur, (3) the presence of cellulitis within the tissue that would become the posterior flap after amputation, and (4) the presence of osteomyelitis at the knee joint, which could extend further into the femur than initially anticipated.

## Conclusions

This case discusses the complicated clinical course of a female with significant comorbidities that ultimately underwent an AKA due to failed knee revisions secondary to the development of multiple PJIs. While there is a gold standard treatment for PJIs, it becomes less clear in the setting of multiple infections and can be highly variable depending on patient factors. Her clinical course required a multidisciplinary approach between infectious diseases, orthopedics, wound care, and the vascular team. It also discusses a unique and complex surgical and medical history. This case aims to add to existing literature about the treatment of complicated and recurrent PJIs as well as highlight a unique patient presentation.
